# New variants and in silico analyses in GRK1 associated Oguchi disease

**DOI:** 10.1002/humu.24140

**Published:** 2020-11-30

**Authors:** James A. Poulter, Molly S. C. Gravett, Rachel L. Taylor, Kaoru Fujinami, Julie De Zaeytijd, James Bellingham, Atta Ur Rehman, Takaaki Hayashi, Mineo Kondo, Abdur Rehman, Muhammad Ansar, Dan Donnelly, Carmel Toomes, Manir Ali, UK Inherited Retinal Disease Consortium, Genomics England Research Consortium, Elfride De Baere, Bart P. Leroy, Nigel P. Davies, Robert H. Henderson, Andrew R. Webster, Carlo Rivolta, Christina Zeitz, Omar A. Mahroo, Gavin Arno, Graeme C. M. Black, Martin McKibbin, Sarah A. Harris, Kamron N. Khan, Chris F. Inglehearn

**Affiliations:** ^1^ Division of Molecular Medicine, Leeds Institute of Medical Research University of Leeds Leeds UK; ^2^ School of Molecular and Cellular Biology, University of Leeds Leeds UK; ^3^ Division of Evolution and Genomic Sciences, School of Biological Sciences, Faculty of Biology, Medicines and Health University of Manchester Manchester UK; ^4^ National Institute of Sensory Organs, National Hospital Organization Tokyo Medical Centre Tokyo Japan; ^5^ Moorfields Eye Hospital London UK; ^6^ UCL Institute of Ophthalmology London UK; ^7^ Keio University School of Medicine Tokyo Japan; ^8^ Ghent University Ghent Belgium; ^9^ Division of Genetic Medicine, Centre Hospitalier Universitaire Vaudois (CHUV) University of Lausanne Lausanne Switzerland; ^10^ The Jikei University School of Medicine Tokyo Japan; ^11^ Mie University Graduate School of Medicine Mie Japan; ^12^ Department of Genetics, Faculty of Science Hazara University Mansehra Dhodial Pakistan; ^13^ Clinical Research Center, Institute of Molecular and Clinical Ophthalmology Basel (IOB) Basel Switzerland; ^14^ School of Biomedical Sciences, University of Leeds Leeds UK; ^15^ Children's Hospital of Philadelphia Philadelphia Pennsylvania USA; ^16^ St Thomas's Hospital London UK; ^17^ Department of Ophthalmology Great Ormond Street Hospital London UK; ^18^ Department of Genetics and Genome Biology University of Leicester Leicester UK; ^19^ Clinical Research Center, Institute of Molecular and Clinical Ophthalmology Basel (IOB) Basel Switzerland; ^20^ Department of Ophthalmology University Hospital Basel Basel Switzerland; ^21^ Sorbonne Université INSERM, CNRS, Institut de la Vision Paris France; ^22^ Manchester Centre for Genomic Medicine, Saint Mary's Hospital, Manchester University NHS Foundation Trust Manchester UK; ^23^ Leeds Teaching Hospitals NHS Trust, St James’ University Hospital Leeds UK; ^24^ School of Physics and Astronomy, University of Leeds Leeds UK

**Keywords:** CSNB, GRK1, Oguchi disease, rhodopsin

## Abstract

Biallelic mutations in G‐Protein coupled receptor kinase 1 (GRK1) cause Oguchi disease, a rare subtype of congenital stationary night blindness (CSNB). The purpose of this study was to identify disease causing GRK1 variants and use in‐depth bioinformatic analyses to evaluate how their impact on protein structure could lead to pathogenicity. Patients’ genomic DNA was sequenced by whole genome, whole exome or focused exome sequencing. Disease associated variants, published and novel, were compared to nondisease associated missense variants. The impact of *GRK1* missense variants at the protein level were then predicted using a series of computational tools. We identified twelve previously unpublished cases with biallelic disease associated GRK1 variants, including eight novel variants, and reviewed all *GRK1* disease associated variants. Further structure‐based scoring revealed a hotspot for missense variants in the kinase domain. In addition, to aid future clinical interpretation, we identified the bioinformatics tools best able to differentiate disease associated from nondisease associated variants. We identified *GRK1* variants in Oguchi disease patients and investigated how disease‐causing variants may impede protein function in‐silico.

## Graphical abstract

In this study, Poulter et al. expand the number of mutations in Rhodopsin Kinase (GRK1), associated with Oguchi disease, from 13 to 21. The authors compare disease associated mutations with likely nonpathogenic variants in a range of bioinformatic prediction software. In silico analyses of the mutations, using a homology model, suggest mutations result in one of three potential mechanisms of disease: loss of protein, loss of kinase function or a failure of prenylation leading to mislocalisation of the protein.

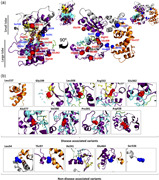

## 1 INTRODUCTION

The first member of the G protein‐coupled receptor kinase (GRK) family was discovered when enzymatic activity was observed in rod membranes that phosphorylated rhodopsin in a light‐dependent manner (Kuhn & Dreyer, 1972). The enzyme, now known as GRK1 (MIM# 180381), was found to be essential for quenching and recycling light‐activated rhodopsin. To be recycled, light‐activated rhodopsin is phosphorylated multiple times by GRK1, followed by binding of Arrestin‐1 to activated‐phosphorylated rhodopsin to block further transducin activation by steric exclusion (Krupnick et al., 1997; Wilden et al., 1986). Failure of this process results in a build‐up of activated rhodopsin, which continues to activate the phototransduction cascade, shutting off circulating current in the rod photoreceptors, rendering them unable to respond electrically to light. Importantly, a build‐up of activated rhodopsin appears to be well tolerated by photoreceptors, with no obvious cell death or structural consequences in the retina, meaning any future therapies leading to the deactivation of rhodopsin could restore visual acuity. Activated cone opsin is likely to be more reliant on GRK7 than GRK1 phosphorylation, as although both enzymes are expressed in cones, loss of GRK1 function has minimal impact on human photopic vision.

**Figure 1:**
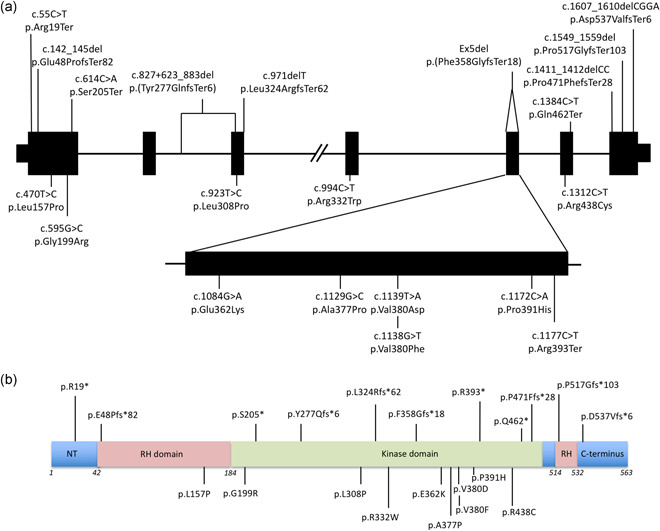
Distribution of disease associated variants within GRK1. All variants shown are annotated according to human genome GRCh37/hg19, using GRK1 gene and protein accession numbers NM_002929 and NP_002920, respectively. Loss of function variants are shown above the gene/protein and missense variants are given below. (a) Genomic organization of *GRK1* showing the location of all novel and published Oguchi disease causing variants. (b) Domain structure of GRK1 showing the location of all GRK1 variants. The start and end amino‐acid positions of each domain are based on Lodowski et al. (2006). NT, *N*‐terminus; RH, regulator of G‐protein signaling homology

GRK1 is a serine/threonine protein kinase with a central catalytic AGC protein kinase (PK) domain that sits within a regulator of G protein signaling homology (RH) domain (Figures 1 and 3). The PK domain binds ATP and the polypeptide substrate which is subsequently phosphorylated (Arencibia et al., 2013). The RH domain is thought to have a key role in receptor binding, with loss of this domain preventing binding and phosphorylation of rhodopsin (He et al., 2017). While the N‐terminus encodes a short alpha‐helical domain, the C‐terminus encodes a lipid‐binding region, which is crucial for prenylation‐dependent docking of GRK1 into the outer segment membranes of rod photoreceptors (Komolov & Benovic, 2018).

**Figure 2:**
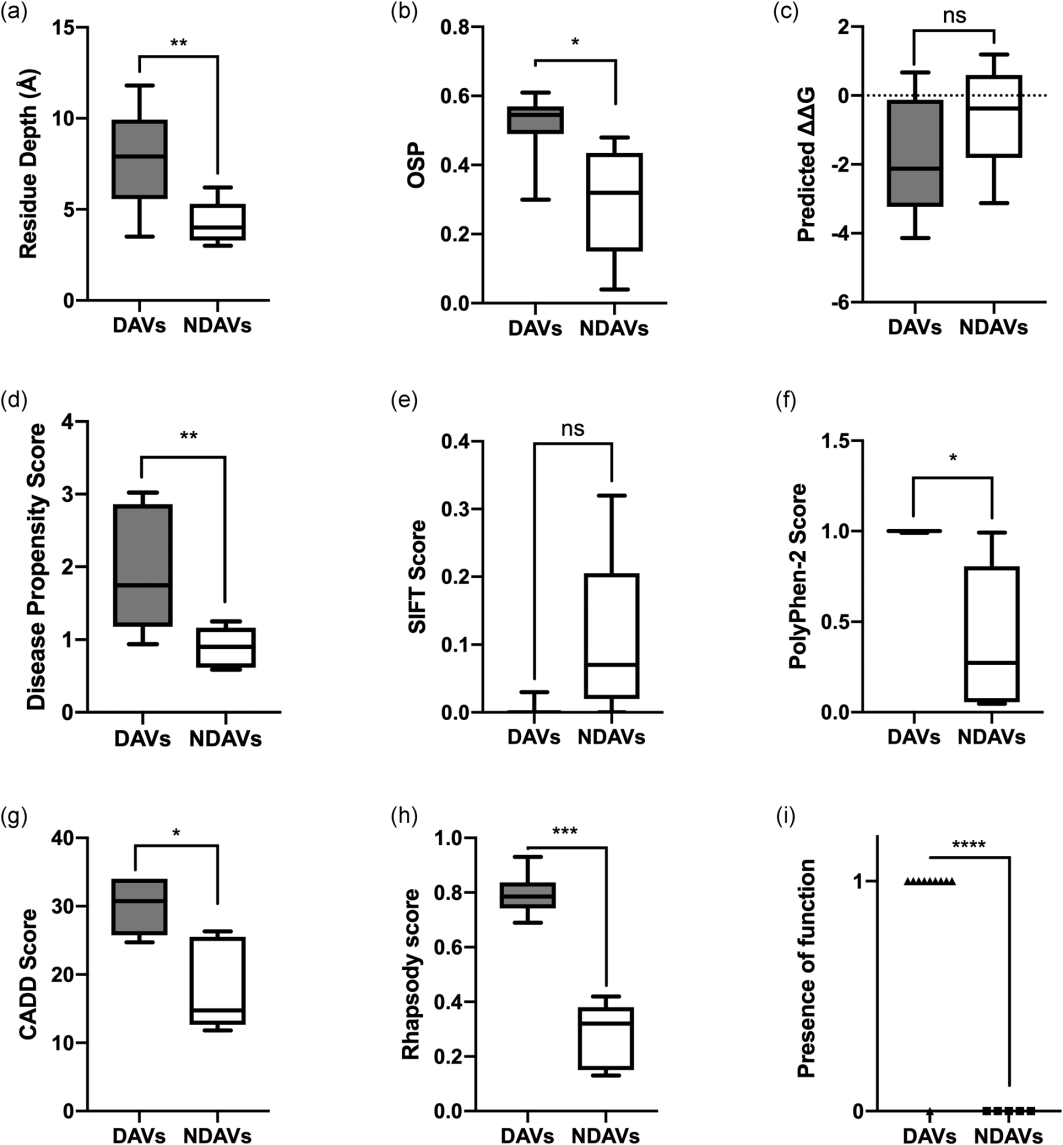
Bioinformatic prediction scores of disease associated and nondisease associated GRK1 variants. Comparison of predicted pathogenicity scores for all novel or published disease associated missense GRK1 variants, with likely nonpathogenic missense variants identified in gnomAD. The whiskers in the box plots show the minimum and maximum values. Statistical significance was calculated using Welch's *t*‐test in GraphPad prism 8.0.2. *ns* = *p* ≥ .05, * = *p* < .05, ** = *p* < .01, *** = *p* < .001, **** = *p* < .0001. (a) Wildtype (WT) residue depth (Å) from site‐directed mutator (SDM), (b) WT occluded surface packing (OSP) from SDM, (c) thermostability score SDM, (d) disease propensity scores as predicted in VarSite, (e) SIFT scores, (f) PolyPhen‐2 scores, (g) CADD (v1.3) scores, (h) Rhapsody scores and (i) ConSurf predicted roles (1 = structural/functional role predicted, 0 = no role predicted). DAVs, disease associated variants; NDAVs, nondisease associated variants

**Figure 3:**
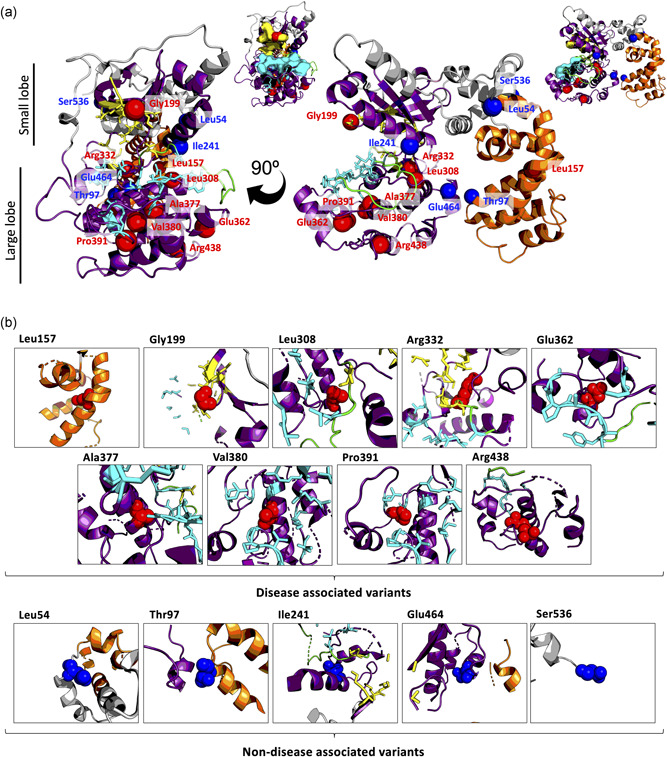
– Location of disease associated and nondisease associated variants in a homology model of GRK1. Homology model of human GRK1 based upon bovine GRK1 structure (PDB: 4PNI). Wildtype residues of disease associated variants are labeled in red (Leu157, Gly199, Leu308, Arg332, Glu362, Ala377, Val380, Pro391, and Arg438) and nondisease associated variants in blue (Leu54, Thr97, Ile241, Glu464, and Ser536). The protein kinase domain is colored in purple, RH domain in orange, activation loop in green, ATP binding residues are represented as yellow sticks and polypeptide substrate binding residues as cyan sticks. (a) A visualization of variant location in the overall structure. Variants are highlighted as beads. The smaller representations to the right, with the ATP and polypeptide binding surface displayed demonstrate how intramolecular residue changes may deform the binding site surface. (b) A focus on residue location and local interactions, displaying only features within 15 Å of the residue of interest. Variant residue atoms are represented as spheres

Oguchi disease (MIM# 613411), a rare form of congenital stationary night blindness (CSNB), results from biallelic variants in either *SAG* (encoding Arrestin‐1) or *GRK1*. *SAG*‐mediated disease is most prevalent in Japanese patients, whilst pathogenic variants in *GRK1* are more common in South East Asians. Typically, Oguchi disease is characterized clinically by the Mizuo–Nakamura phenomenon—the presence of a golden‐yellow colouration to the retina that disappears in the dark‐adapted state and reappears shortly after light exposure (Miyake et al., 1996). Electrophysiologically there is normal cone function, delayed rod dark adaptation and marked rod desensitization to a bright flash. Most reported cases have this distinctive phenotype and no significant variation in disease expression has been reported, with the exception of a few rare cases (Hayashi et al., 2007; Nishiguchi et al., 2019). Distinguishing between Oguchi disease and other forms of CSNB is important as they may have different prognoses—some patients with Oguchi disease report very slowly progressive visual dysfunction, whereas those with classical CSNB do not. Minimally progressive retinal degeneration has been observed in *SAG*‐associated Oguchi disease, and for one patient, this resulted in their disease being re‐classified as retinitis pigmentosa 26 years after being diagnosed with a stationary rod dysfunction syndrome (Oguchi disease; Nishiguchi et al., 2019). As most of our knowledge of Oguchi disease stems from our understanding of loss of SAG function further research is required to better understand *GRK1*‐related Oguchi disease.

To date, thirteen pathogenic variants in *GRK1* have been implicated in Oguchi disease, eight of which are predicted to be null variants (Azam et al., 2009; Cideciyan et al., 1998; Godara et al., 2012; Hayashi et al., 2007; Jespersgaard et al., 2019; Li et al., 2017; Mucciolo et al., 2018; Oishi et al., 2007; Skorczyk‐Werner et al., 2015; Teke et al., 2016; Yamamoto et al., 1997; Q. Zhang et al., 2005). The molecular mechanisms by which these contribute to disease are poorly understood. Here, we present eight further disease associated, likely pathogenic variants in *GRK1* and review new and known variants causing Oguchi disease. Using in‐silico techniques we compare likely pathogenic disease‐associated variants with nonpathogenic *GRK1* missense variants to identify features that could be contributing to the phenotype. Together this will help define disease mechanisms and assist in predicting likelihood of pathogenicity for variants identified in *GRK1*.

## 2 METHODS

### 2.1 Editorial policies and ethical considerations

Ethical approval for this study was obtained from the Yorkshire and The Humber – Leeds East Research Ethics Committee (REC reference: 17/YH/0032). The study adhered to the tenets of the Declaration of Helsinki. Blood samples were taken with informed consent from each participant, or with parental informed consent on behalf of children.

### 2.2 Clinical assessment

Study participants were ascertained from St James’ University Hospital, Leeds, England; Manchester Centre for Genomic Medicine, Manchester, England; Moorfields Eye Hospital, London, England; Department of Ophthalmology, Ghent University Hospital, Ghent, Belgium; The Jikei University School of Medicine, Tokyo, Japan; Mie University Graduate School of Medicine, Mie, Japan; and National Institute of Sensory Organs, National Hospital Organization Tokyo Medical Centre, Tokyo Japan; individuals were recruited following clinical examination by an experienced ophthalmologist, with the exception of Families 10 and 11 who were ascertained by local doctors in Pakistan.

### 2.3 Sequencing

Exome and clinical exome sequencing was performed on 3 µg of genomic DNA using the Agilent SureSelectXT Human All Exon (V6) and Focussed Exome respectively (Agilent Technologies), according to the manufacturer's instructions. Captured libraries were pooled and sequenced on an Illumina HiSeq3000 sequencer (Illumina). The resulting fastq files were aligned to the Human genome assembly GRCh37 using Burrows‐Wheeler Aligner and reads processed using samtools, Picard, and the Genome Analysis Toolkit. Variants within the captured regions were called and annotated using variant effect predictor (NCBI) and further filtered for those within genes known to cause retinal disease. This analysis pipeline is described in more detail elsewhere (Panagiotou et al., 2017). Copy number variation analysis was performed using ExomeDepth, comparing patient BAM files with BAM files from unrelated individuals in the same sequencing run. All novel variants were confirmed by Sanger sequencing using standard methods.

The affected individual and unaffected parents of Family 1 underwent Genome sequencing as part of the 100,000 Genome Project, as previously detailed by Taylor et al. (2017).

### 2.4 Identifying nonpathogenic variants

To identify protein‐level characteristics influencing a disease phenotype we compared the nine disease associated missense variants with a group of five assumed nondisease associated missense variants. For the nonpathogenic variants, we identified all homozygous *GRK1* missense variants in gnomAD and only included those with a MAF>0.00035, the level above which variants are calculated to be too common to cause Oguchi disease (Whiffin et al., 2017). The five missense variants in gnomAD with frequencies above this level, p.Leu54Phe, p.Thr97Met, p,Ile241Thr, p.Glu464Gln, and p.Ser536Leu, also all occur at least once as homozygotes in gnomAD. These are therefore assumed to be nondisease associated polymorphisms (further details in Table S1).

### 2.5 Structural analysis

A homology model of human wild‐type GRK1 based on the bovine structure (PDB: 4PNI) was produced using SwissModel (Waterhouse et al., 2018). The locations of the RH and PK domains, ATP binding site, polypeptide substrate binding site and activation loop were identified using InterPro (Mitchell et al., 2019). The structure was visualized, and regions of interest labeled using PyMOL (Schrodinger, 2015). Residue depth and connectivity within the structure was determined using Site‐Directed Mutator (SDM; Pandurangan et al., 2017). To further explain variant impact, changes in residue physical characteristics; molecular weight, isoelectric point, and hydrophobicity (Laskowski et al., 2020) were reviewed.

### 2.6 Scoring missense variants

A number of bioinformatics tools were reviewed to identify which approach best classified the variants. Scoring methods included: SDM (Pandurangan et al., 2017), VarSite (Laskowski et al., 2020), SIFT v6.2.1 (Sim et al., 2012), Polyphen‐2 v2.2.2r394 (Adzhubei et al., 2010), CADD v1.3 (Kircher et al., 2014), Rhapsody (Ponzoni et al., 2019), and ConSurf (Ashkenazy et al., 2016). All statistical analyses were performed in GraphPad Prism 8.0.2 (for macOS, GraphPad Software, www.graphpad.com) using Welch's *t*‐test.

## 3 RESULTS

### 3.1 Identification of novel *GRK1* variants associated with Oguchi disease

Screening of inherited retinal disease patients by an international consortium of retinal genetics laboratories identified 12 cases with Oguchi disease harboring nine different disease associated variants in *GRK1*, of which eight are previously unreported (Table 1). The majority of cases in this study were identified in families of South Asian descent, except for Family 2 who are East Asian (Japanese) and Family 5 who are Eastern European (Polish). All variants were confirmed and segregated in available family members (summarized in Figure S1). Each patient had a history of non/minimally progressive night blindness, and retinal examination revealed the Mizuo–Nakamura phenomenon, consistent with a clinical diagnosis of Oguchi disease.

**Table 1:** Summary of all novel and published mutations in GRK1

DNA variant	Exon	Protein variant	rs number	gnomAD Frequency	Domain	PolyPhen‐2	CADD (v1.3)	References
c.55C>T	1	p.Arg19Ter	rs370713047	17/239,700 (1 homozygous)	N‐Terminus	N/A	37.0	Li et al. (2017)
c.142_145del	1	p.Glu48ProfsTer82	rs748680704	6/248,384	RH domain	N/A	28.5	This study (**F1, F8, F11**)
c.470T>C	1	p.Leu157Pro	N/A	0	RH domain	1.00 (Prob. D)	26.0	Mucciolo et al. (2018)
c.595G>C	1	p.Gly199Arg	N/A	0	Kinase	1.00 (Prob. D)	24.7	This study (**F2**)
c.614C>A	1	p.Ser205Ter	N/A	0	Kinase	N/A	36	Azam et al. (2009)
c.827+623_883del	3	p.(Tyr277GlnfsTer6)	N/A	0	Kinase	N/A	27.8	Zhang et al. (2005)
c.923T>C	3	p.Leu308Pro	N/A	0	Kinase	0.996 (Prob. D)	29.2	Teke et al. (2016)
c.971del	3	p.Leu324ArgfsTer62	N/A	0	Kinase	N/A	35	Oishi et al. (2007)
c.994C>T	4	p.Arg332Trp	rs372114657	0	Kinase	1.000 (Prob. D)	29.5	This study **(F12)**
Ex5 del	5	p.(Phe358GlyfsTer18)	N/A	0	Kinase	N/A	25.1^a^	Yamamoto et al. (1997); Cideciyan et al. (1998)
c.1084G>A	5	p.Glu362Lys	N/A	0	Kinase	1.00 (Prob. D)	34	This study (**F3**)
c.1129G>C*	5	p.Ala377Pro	N/A	0	Kinase	0.991 (Prob. D)	25	Godara et al. (2012)^1^
c.1138G>T	5	p.Val380Phe	N/A	2/173,226	Kinase	0.998 (Prob. D)	32	This study (**F4**)
c.1139T>A*	5	p.Val380Asp	rs777094000	5/173,124	Kinase	0.999 (Prob. D)	34	Yamamoto et al. (1997)^2^; Godara et al. (2012)^1^
c.1172C>A	5	p.Pro391His	rs570621429	1/141,802	Kinase	1.00 (Prob. D)	34	Hayashi et al. (2007)
c.1177C>T	5	p.Arg393Ter	rs137877289	6/141,764	Kinase	N/A	46	This study (**F5**)^3^
c.1312C>T	6	p.Arg438Cys	rs765070399	2/141,922	Kinase	1.00 (Prob. D)	34	This study (**F9**)
c.1384C>T	6	p.Gln462Ter	N/A	0	Kinase	N/A	42	Jespersgaard et al. (2019)
c.1411_1412del	7	p.Pro471PhefsTer28	N/A	0	Kinase	N/A	35	Oishi et al. (2007)
c.1549_1559del	7	p.Pro517GlyfsTer130	N/A	0	RH domain	N/A	35	This study (**F6**)
c.1607_1610del	7	p.Asp537ValfsTer7	rs756235051	89/172,958	C‐Terminus	N/A	26.7	Yamamoto et al. (1997)^2^; Skorczyk‐Werner et al. (2015); This study (**F5** ^3^,**F7, F10**)

*Note*: Lodowski et al. (2006). All variants identified are all homozygous except for ^1,2,3^ which are compound heterozygous,

* Predicted – DNA mutation not provided in publication, Prob. D = Probably Damaging as scored by PolyPhen2.

^a^ Based on deletion of whole of exon 5 and surrounding splice sites as exact coordinates are unknown.

A literature search revealed a further thirteen published Oguchi disease causing *GRK1* variants from 12 studies (Azam et al., 2009; Cideciyan et al., 1998; Godara et al., 2012; Hayashi et al., 2007; Jespersgaard et al., 2019; Li et al., 2017; Mucciolo et al., 2018; Oishi et al., 2007; Skorczyk‐Werner et al., 2015; Teke et al., 2016; Yamamoto et al., 1997; Q. Zhang et al., 2005). The nomenclature for these variants was updated based on the current HGVS nomenclature guidelines and the GRCh37 version of the Human Genome, and together with the new variants reported here, these have been included in a Leiden Open Variation Database (LOVD) of *GRK1* variants (http://dna2.leeds.ac.uk/GRK1_LOVD/genes/GRK1).

### 3.2 Disease associated variant distribution and domain structure

The 21 Oguchi disease associated variants (DAVs) identified to date include missense (*n* = 10), frameshift (*n* = 6), and nonsense (*n* = 4) variants (Table 1) as well as a large deletion encompassing exon 5 (Figure 1a). Each variant has been identified in only a single family with the exception of p.Val380Asp (two families), p.Glu48ProfsTer82 (two families), the deletion of exon 5 (p.(Phe358GlyfsTer18); three families), and the C‐terminal frameshift variant p.(Asp537ValfsTer6; six families), with the latter variant having the highest allele frequency in gnomAD (89/172,958). Of the 89 p.Asp537ValfsTer7 alleles in gnomAD, 42 are in European (non‐Finnish) and 35 in South Asian populations, which is consistent with this mutation being identified in cases from Eastern Europe and South Asia in this study.

Sixteen of the variants lie within the protein kinase domain, three within the RH domain and one at each of the N‐ and C‐termini (Figure 1b). While the frameshift and nonsense variants are present throughout the protein, all except one of the missense variants (*n* = 8 out of 9) are present within the protein kinase domain (Figure 2a), and five cluster within a 30 amino‐acid region between Glu362 and Pro391 (Figure 2a). These are likely to impair phosphorylation of light‐activated rhodopsin.

Three variants located after this cluster introduce frameshifts in the final exon, giving rise to transcripts which would not be expected to undergo nonsense‐mediated decay (Thermann et al., 1998). The extreme C‐terminus of GRK1, which these three variants would disrupt, is prenylated to facilitate anchoring of GRK1 in the membranes of the rod photoreceptor outer segments (Pitcher et al., 1998), which maximizes the likelihood of interaction with activated rhodopsin. Lack of this domain is therefore likely to reduce the level of anchored GRK1, which will in turn reduce the quantity of rhodopsin being bound and phosphorylated.

### 3.3 Disease associated missense variants affect key regions in *GRK1* active site

To further understand the mechanism by which the missense variants affected GRK1 function, we performed structure‐based scoring on the SDM server using our homology model (Figure 2a–c). Although SDM's primary function is predicting thermal stability, the most interesting results came from the scoring of wildtype (WT) residue depth and occluded surface packing (OSP), with both scores showing a significant difference between DAVs and likely non‐pathogenic, nondisease associated variants (NDAVs) (*p* = .0021 and *p* = .0390 respectively). This demonstrated that DAVs were located deeper within the three‐dimensional (3D) structure of *GRK1* than NDAVs (residue depth = 3.5–11.8 and 3–6.2, respectively; Figure 2a, Table S2) and were more connected/densely packed (OSP = 0.3–0.61 and 0.04–0.48, respectively; Figure 2b, Table S2). Exceptions to these rules were seen in DAV p.Gly199Arg, which is located at the surface of the protein (residue depth = 3.5 Å and OSP = 0.3), and NDAV p.Ile241Thr, which is located deeper in GRK1 (residue depth = 6.2 and OSP = 0.48). Gly199 is positioned within the conserved glycine rich loop of the PK domain and is part of the ATP binding site (Figure 3). Mutation to a bulky positively charged arginine (Table S2) is likely to disrupt both the shape and charge of the ATP binding pocket (Figure 3). Although Ile241 is buried in the binding pocket of the PK domain (Figure 3), there is little change in size and charge (Table S2) which could explain why this variant is nonpathogenic.

Further investigation of mutation location in the protein structure demonstrated DAVs were located proximal to key sites for protein function, including the substrate and ATP binding domains, while NDAVs were not (Figure 3a). DAVs were predicted to be likely to disrupt the shape and charge of the active site either directly as in the case of p.Gly199Arg and p.Arg332Trp, or allosterically through introduction of proline kinks into alpha helices (e.g., p.Leu308Pro and p.Ala377Pro), intramolecular charged groups (classically exposed; e.g., p.Val380Asp and p.Pro391His), and bulky side groups within densely packed regions (e.g., p.Val380Phe) close to key residues (Figure 3b and Table S2). In the case of p.Arg438Cys and p.Glu362Lys, the equivalent arginine and glutamic acid residues are conserved and form a salt bridge in the bovine protein (Figure S2). It is therefore likely that substitution of Glu362 and Arg438 changes the charge of these residues in the human protein and is disrupting this salt bridge interaction (Figure S2). Meanwhile NDAVs were often subtler changes, and not local to key residues (Figure 3b and Table S2). We hypothesize DAVs within the protein kinase domain are inhibiting protein function through disruption of the shape and dynamics of this key region, while NDAVs prove less disruptive to shape as they are not internal and/or have subtler changes in residue characteristics. This is supported by Consurf's prediction that most of the DAVs occur at loci important for protein structure or function (Figure 2i and Table S2).

Only one DAV, p.Leu157Pro, lies outside of the kinase domain, instead being present within the RH domain of GRK1, the domain primarily responsible for rhodopsin binding (He et al., 2017). The introduction of a proline residue within an α‐helix is likely to disrupt the secondary structure and introduce a characteristic “kink” due to its rigidity and constrained phi angle. This could potentially impact on the ability of Rhodopsin to correctly bind GRK1 in order for phosphorylation to occur.

To determine which bioinformatic tools were most successful in differentiating between disease associated and nondisease associated variants, we scored missense variants using: VarSite, SDM, PolyPhen‐2, SIFT, CADD, Rhapsody, and Consurf. Predictions were compared between DAV and NDAV sets, with significance assessed by Welch's *t*‐test (Figure 2, Tables 1, and S2). VarSite disease propensity, WT OSP, WT residue depth, PolyPhen‐2, CADD, Rhapsody, and Consurf, were able to distinguish between DAVs and NDAVs with statistical significance. Consurf's structure/function prediction and Rhapsody, which both take the variant's location in the protein 3D structure into consideration, were able to differentiate between disease associated and nondisease associated most successfully (*p* < .0001; Figure 2h,i and Table S2).

**Figure 4:**
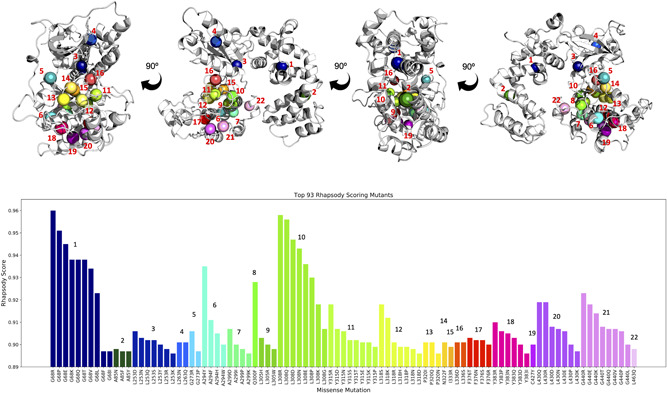
– Rhapsody top scoring substitutions mapped onto the homology model of GRK1. Homology model of human GRK1 based upon bovine GRK1 structure (PDB: 4PNI). Wildtype residues of top‐scoring ((≥0.896) Rhapsody variants are highlighted as beads, and labeled, colored and numbered based on residue number. The colors and labels within the bar chart of top‐scoring Rhapsody variants mimic this format. Locations with more than four variants in the top 93 scores include: G68, L253, L308, Y315, L318, Y383, L430, and G440

Rhapsody has the additional feature of being able to predict the impact of substituting any residue in a protein with all of the 19 other amino acids (in silico site‐directed mutagenesis), making it extremely valuable diagnostically, as it has the potential to inform future work by identifying residue changes which are likely to be poorly tolerated. We performed protein‐wide site‐directed mutagenesis and reviewed the top scoring variants (Rhapsody score ≥ 0.896). This analysis revealed that the top scoring variants were present at just 22 loci across the protein, thus identifying a number of key residues with the potential to cause Oguchi disease, if mutated. Of the 22 amino acid residues that Rhapsody predicts could harbor the most damaging variants, 20 are located in the protein kinase domain (Figure 4).

## 4 DISCUSSION

Using a combined genetics and structural biology approach we have identified individuals with Oguchi disease due to biallelic *GRK1* variants and inferred the likely functional consequences of these and other published variants. We identified eight new disease associated variants, taking the total number of *GRK1* variants associated with Oguchi disease to 21. While eleven result in premature termination codons, 10 result in single amino acid substitutions.

It is likely that transcripts encoding nonsense and frameshift variants, except those in the last exon, will be subject to nonsense mediated decay (NMD; Thermann et al., 1998). This has not been confirmed in patient cells, but even if a truncated GRK1 was expressed, previous studies have shown that GRK1 protein lacking the residues encoded by exon 5 had significantly reduced function, resulting in protein that was not able to phosphorylate Rhodopsin (Cideciyan et al., 1998). This implies that lack of functional GRK1 protein is one mechanism by which Oguchi disease can arise.

Eight of the 10 observed missense variants, and 20 of the 22 top Rhapsody scorers lie within the protein kinase domain, which is critical for GRK1 function. The kinase domain is the largest within GRK1, and variants in this region are likely to have a negative impact on phosphorylation of light‐activated rhodopsin. Indeed, a previous study has shown the p.Val380Asp variant has no kinase activity for Rhodopsin compared with wild‐type GRK1 (Khani et al., 1998). Five missense variants appear to be clustered within a 30 amino‐acid region between Glu362 and Pro391, which may imply that amino‐acid substitutions in this region are more likely to disrupt protein function than those located elsewhere. These variants were predicted to affect the structure of GRK1 and therefore kinase activity. As the structural analysis relies on a homology model of human GRK1, it is not always feasible to quantify atomic level changes (e.g., the formation of novel H‐bond or salt bridge interactions, or the disruption of interactions within non‐conserved residues). However, it is possible to draw general conclusions about the surrounding residue location and impact on secondary and tertiary structure. Therefore, loss of kinase activity through disruption of its shape and dynamics is a second mechanism by which Oguchi disease can occur.

Three variants have been identified in the last exon of *GRK1*, all of which result in frameshift transcripts that potentially escape nonsense mediated decay, leading to a GRK1 protein with a disrupted C‐terminus. This region plays a crucial role in embedding GRK1 within the photoreceptor outer segment membrane; GRK1 localizes directly to the outer segment membranes due to a short prenylation sequence within the C‐terminus that interacts with the prenyl‐binding protein, delta (PrBP/δ), which facilitates attachment to the membranes (Huang et al., 2011; Roosing et al., 2014). Deletion of PrBP/δ has also been shown to result in impaired trafficking of prenylated‐GRK1 to photoreceptor outer segments, highlighting the importance of this interaction in correct localization of GRK1 (H. Zhang et al., 2007). This cellular localization is essential for GRK1 function as it brings active rhodopsin into contact with docked GRK1 protein through a two‐dimensional search rather than 3D diffusion, which is essential for ultrarapid signal termination (Sato et al., 2015). Lack of this sequence is likely to cause failure of prenylation, leading to abolished binding of PrBP/δ, incorrect localization of GRK1 and a reduction in Rhodopsin phosphorylation, a third mechanism by which disease can arise. This is supported by studies showing a marked reduction in Rhodopsin phosphorylation in cells transfected with the p.Asp537ValfsTer7 variant (Khani et al., 1998).

One missense variant, p.Leu157Pro, was identified in the N‐terminal RH domain, which of the two RH domains present in GRK1, contains most of the Rhodopsin binding capacity (~70%). While complete loss of the N‐terminal RH domain has been shown to cause a significant reduction in Rhodopsin binding (He et al., 2017), no studies have yet determined the effect of single amino acid substitutions on this activity. The importance of Leu157, however, is highlighted by it being conserved across all known human GRKs, suggesting it is crucial for the function of the protein. Binding of Rhodopsin has also been shown to require the protein kinase domain to be in an active conformation (Homan & Tesmer, 2014), therefore missense substitutions in the kinase domain may also have an impact on Rhodopsin binding. Further studies are required to determine the full extent of missense substitutions on GRK1 function.

Our study therefore highlights three potential molecular mechanisms by which GRK1 variants lead to disease, each of which ultimately reduce or abolish the phosphorylation of light‐activated rhodopsin. These are (a) lack of GRK1 protein due to frame shift or nonsense variants in all exons but the last, resulting in NMD; (b) missense variants leading to inability of GRK1 to phosphorylate rhodopsin due to a dysfunctional kinase domain or failure to bind rhodopsin, ATP or Mg^2+^; or (c) a frameshift in the final exon, which would be expected to escape nonsense mediated decay and produce a protein retaining the ability to bind and phosphorylate light‐activated rhodopsin, but which cannot localize to the outer segment membranes. We would therefore predict that, while variants in the first category lead to no protein being produced, those in the second produce proteins that correctly localize within the outer segments but have inhibited kinase activity, and those in the third produce a GRK1 protein likely to maintain kinase activity but which fails to localize and so rarely comes into contact with activated rhodopsin. Interestingly, the kinase activity of the p.(Asp537ValfsTer6) variant was found to be significantly reduced in transfected cos7 cells, suggesting C‐terminal mutations may affect kinase activity as well as protein localization. However, this lack of activity may be explained by difficulties in expressing the mutation containing GRK1 protein rather than on protein function (Khani et al., 1998).

Our comparison of GRK1 DAVs to NDAVs identified from gnomAD facilitated an assessment of pathogenicity scores, which could inform future variant interpretation. We found that VarSite disease propensity, Polyphen2, CADD, Rhapsody and Consurf were able to significantly differentiate between DAVs and NDAVs, as were biophysical scores of residue depth and occluded surface packing. Rhapsody was the best of all the tools at differentiating between DAVs and NDAVs. Rhapsody incorporates dynamics information from elastic network models of protein structure, as well as PolyPhen‐2 and EVMutation (conservation) scores using a machine learning approach. This broad range of sequence‐, structure‐, and dynamics‐based information could account for its success since the impact of a variant can arise from a multitude of different physio‐chemical effects. Despite producing the best separation (Rhapsody) and describing features (SDM), structure‐based tools are limited by the availability of the protein's structure or a homologous structure. Although homology models are not always accurate for atomic level information, coarser detail such as whether residues are buried is likely to be reliable. Similarly, although ConSurf can be used without a pdb structure it is dependent on homologous proteins having a structure. In this study we found using ConSurf without a pdb structure was sufficient to predict pathogenicity based on whether a residue is likely to be buried or exposed, relating to its location in homologous proteins with known structures. Ultimately, these scores can only be used as a guide, and variant pathogenicity should be confirmed by segregation analysis and functional assays wherever possible, as rare nonpathogenic variants can also score highly.

Interrogation of gnomAD revealed a further three rare variants that were homozygous in at least one individual and could not be excluded as a cause of Oguchi disease based on allele frequency alone (Table S3 and Figure S3). We therefore calculated the same set of pathogenic prediction scores to assess the likely pathogenicity of these variants of unknown significance. The p.Met185Val variant, although rare in gnomAD, produced scores that were comparable with the NDAVs for each tool, suggesting it does not contribute to disease. However, both p.Ala353Ser and p.Ala387Val, produced scores that were compatible with DAVs, suggesting that the two individuals in gnomAD that are homozygous for these variants may have Oguchi disease. gnomAD contains exome and genome sequence data from over 130,000 unrelated individuals, with only those individuals affected by a severe pediatric disorder being removed. It is therefore possible that individuals with CSNB or Oguchi disease are present in the gnomAD database which could explain the presence of two homozygous, likely DAVs.

In summary, we present eight novel variants in *GRK1* as a cause of Oguchi disease and perform an in‐depth analysis of all *GRK1* variants to understand their contribution to disease. We describe three mechanisms likely to account for all *GRK1*‐related disease, each of which ultimately result in a failure to phosphorylate light‐activated rhodopsin. We assessed the ability of different in silico pathogenicity scores to differentiate DAVs and NDAVs and found that Rhapsody out‐performed all tools tested for this data set. Finally, we have created an LOVD database into which all the known DAVs and several common NDAVs described herein have been entered, to guide future variant interpretation in *GRK1*‐associated disease.

## CONFLICTS OF INTEREST

All the authors declare that there are no conflicts of interests.

## Supporting information

Supporting information.

Supporting information.

Supporting information.

Supporting information.

## Data Availability

All disease associated GRK1 variants described to date have been collated into a Leiden Open Variation Database (http://dna2.leeds.ac.uk/GRK1_LOVD/genes/GRK1). The variants reported in this paper have been deposited into the ClinVar database (https://www.ncbi.nlm.nih.gov/clinvar/) at the National Center for Biotechnology Information under accession numbers SCV001245346 ‐ SCV001245364. Supplemental Data includes three figures and three tables. The 3D homology model of Human GRK1 based upon the bovine GRK1 crystal structure (PDB: 4PNI) is available on request from the corresponding author. The full data that supports the findings of this study are available in the supplementary material of this article.
