# Supplementary material for: New variants and in silico analyses in GRK1 associated Oguchi disease
Source: Hum Mutat. 2020 Nov 30;42(2):164–76. doi: 10.1002/humu.24140 (PMC7898643; doi:10.1002/humu.24140)

Supplementary Figure 1 – Summary of new families identified in this study. A summary of the twelve new families with a GRK1 variant, describing the genotype, phenotype and segregation where available. 'M' represents mutation containing allele, '+' represents the wildtype allele. Shaded pedigree symbols signify an affected individual. For large pedigrees, a number within a shape is used to signify the total number of individuals of that gender in the sib-ship.

Family 1
Variant: homozygous c.142_145del, p.Glu48ProfsTer82
Origin: Consanguineous, S. Asian Family residing in the UK.
Phenotype: Classical fundus appearance of Oguchi disease.
Nyctalopia onset in early teens.

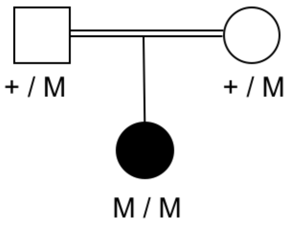

Family 2
Variant: homozygous c.595G>C, p.Gly199Arg
Origin: East Asian family, residing in Japan. No family history of disease.
Phenotype: Classical fundus appearance of Oguchi disease.

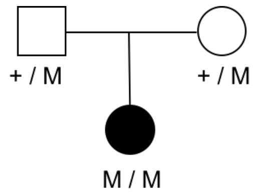

Family 3
Variant: homozygous c.1084G>A, p.Glu362Lys
Origin: Consanguineous, S. Asian family residing in the UK.
Phenotype: Myopia with lifelong poor vision. No nystagmus, excellent Snellen VA, normal CFP and OCT.

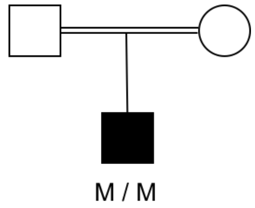

Family 4
Variant: homozygous c.1138G>T, p.Val380Phe
Origin: Consanguineous, S. Asian family residing in the UK.
Phenotype: Classical fundus appearance of Oguchi disease.

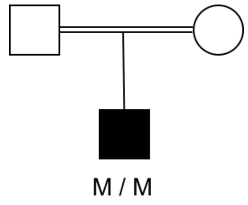

Family 5
Variant: compound heterozygous c.1607_1610del, p.Asp537ValfsTer7 (M1); c.1177C>T, p.Arg393Ter (M2)
Origin: Eastern European (Polish)
Phenotype: Lifelong night blindness with classical fundus appearance of Oguchi disease.

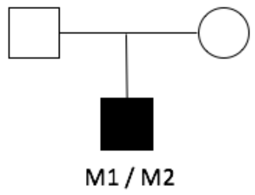

Family 6
Variant: homozygous c.1549_1559del, p.Pro517GlyfsTer130
Origin: South Asian (Indian) Female residing in Belgium.
Phenotype: Classical fundus appearance of Oguchi disease.

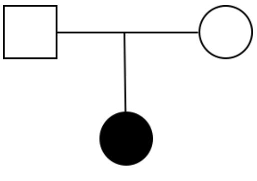

Family 7
Variant: homozygous c.1607_1610del, p.Asp537ValfsTer7
Origin: Unkown
Phenotype: Classical fundus appearance of Oguchi disease.

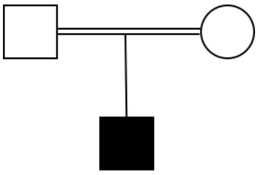

Family 8 (G95556)
Variant: homozygous c.142_145del, p.Glu48ProfsTer82, Manchester
Origin: Consanguineous, S. Asian Family, residing in the UK.
Phenotype: RP & Night blindness. Mid-peripheral and peripheral retina has a 'whitish yellow sheen'. Only reports problems with vision at night but also wears glasses for distance.

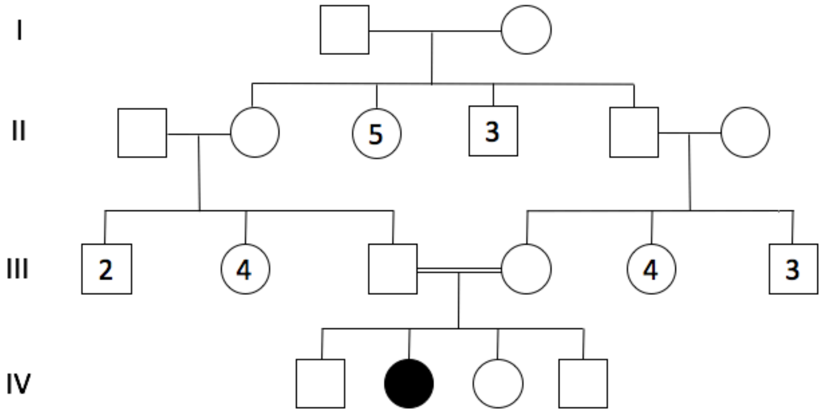

Family 9
Variant: homozygous c.1312C>T, p.Arg438Cys.
Origin: Unknown
Phenotype: Classical fundus appearance of Oguchi disease. Longstanding night blindness. Atypical ERGs (with cone system involvement).

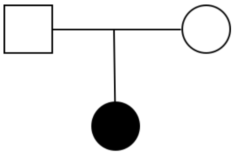

Family 10 (PK404)
Variant: homozygous c.1607_1610del, p.(Asp537ValfsTer7).
Origin: South Asian Family residing in North-West Pakistan.
The variant segregates in all family members labelled with a ‘PK’ number.
Phenotype: Classical fundus appearance of Oguchi disease.

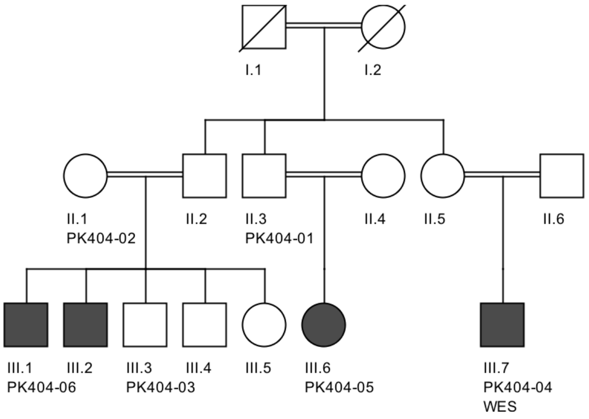

Family 11
Variant: homozygous c.142_145del, p.Glu48ProfsTer82.
Origin: South Asian (Pakistan).
Phenotype: Classical fundus appearance of Oguchi disease.
The variant segregates in all available family members (marked with * in the pedigree)

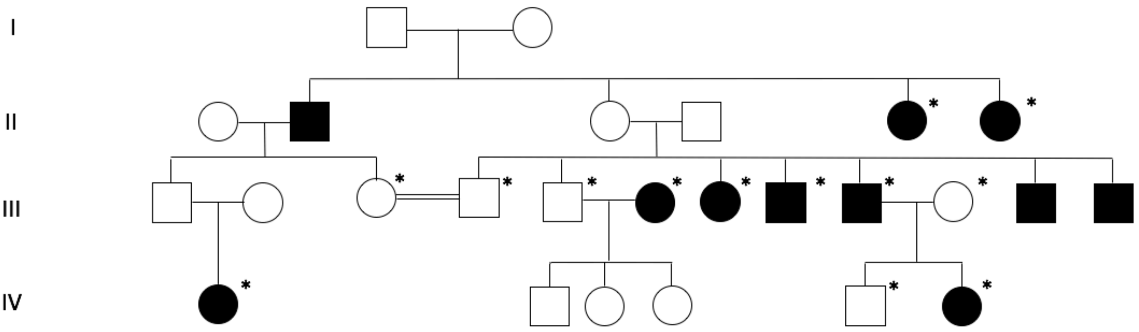

Poulter et al., New pathogenic variants and in-silico analyses in GRK1-related Oguchi disease.
Supplementary Data

Family 12 (MAPK0076_PKIURP05)
Variant: homozygous c.994C>T, p.Arg332Trp.
Origin: South Asian (Pakistan)
Phenotype: Classical fundus appearance of Oguchi disease.
The variant segregates in all samples labelled with *.

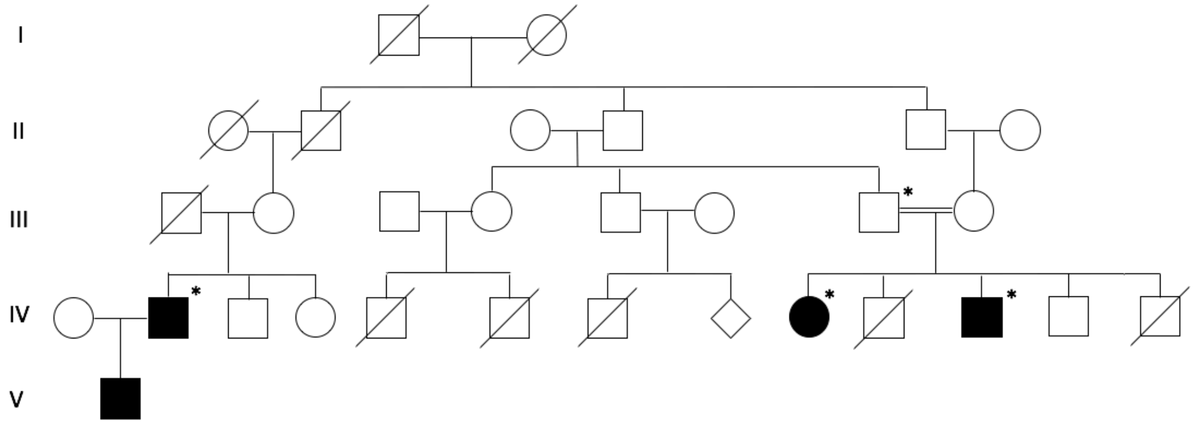

Supplement: Supplementary file 2 — Supporting information. [file HUMU-42-164-s002.pdf]
