# Supplementary material for: New variants and in silico analyses in GRK1 associated Oguchi disease
Source: Hum Mutat. 2020 Nov 30;42(2):164–76. doi: 10.1002/humu.24140 (PMC7898643; doi:10.1002/humu.24140)
Supplement: Supplementary file 3 — Supporting information. [file HUMU-42-164-s003.pdf]

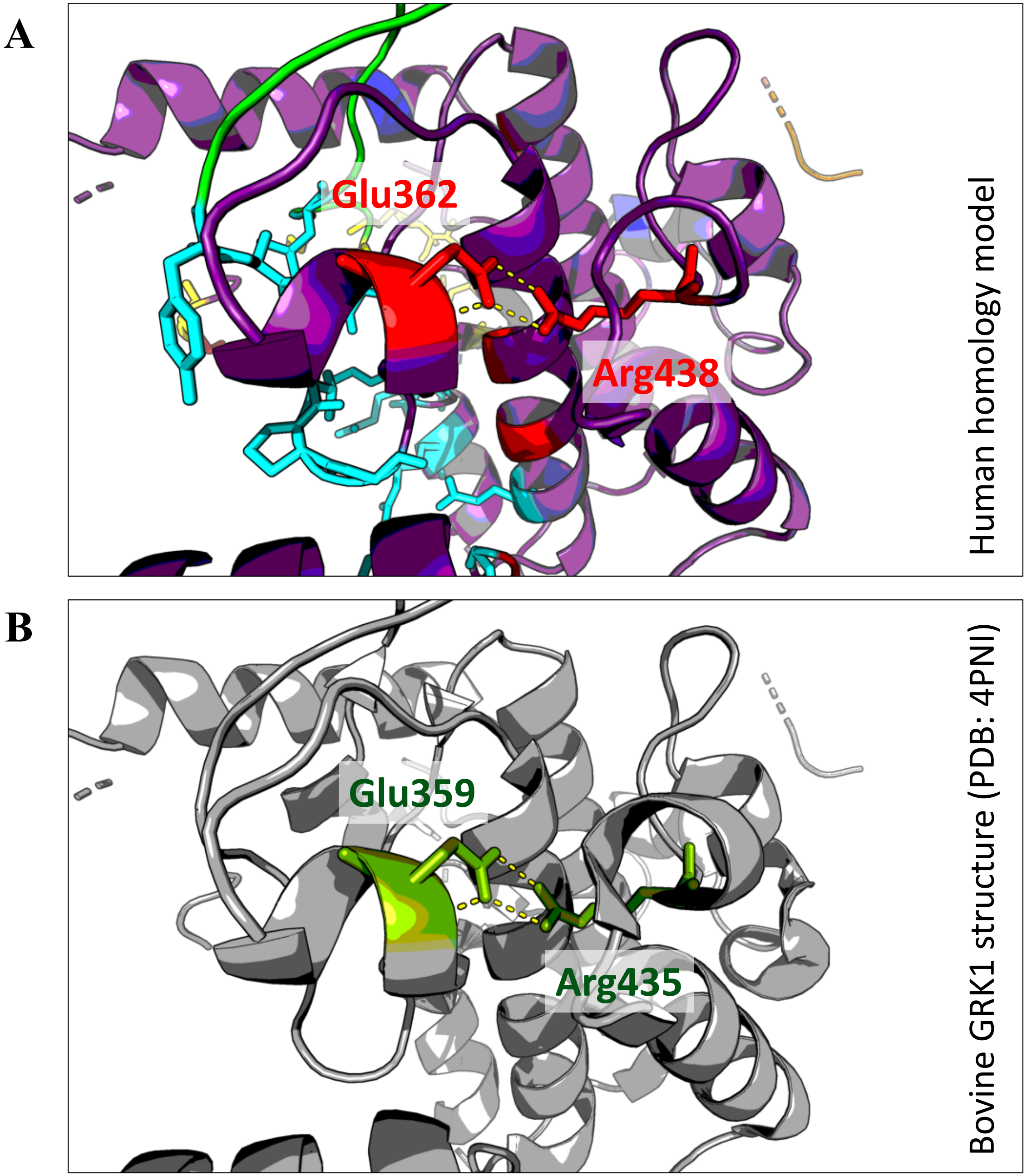

Supplementary Figure 2 – Mutations at Glu362 and Arg438 are likely to affect GRK1 structure through disruption of a salt bridge interaction.

A) Human homology model of GRK1 showing residues Glu362 and Arg438 interacting via a salt bridge. Substitution of either residue has been identified as a cause of Oguchi disease.

B) The homologous region in the bovine GRK1 crystal structure, showing the same residues (Glu359 and Arg435) interacting via a salt bridge.
